# Taxonomic review of *Gasterophilus* (Oestridae, Gasterophilinae) of the world, with updated nomenclature, keys, biological notes, and distributions

**DOI:** 10.3897/zookeys.891.38560

**Published:** 2019-11-21

**Authors:** Xin-Yu Li, Thomas Pape, Dong Zhang

**Affiliations:** 1 School of Ecology and Nature Conservation, Beijing Forestry University, Qinghua east road 35, Beijing 10083, China Beijing Forestry University Beijing China; 2 Natural History Museum of Denmark, University of Copenhagen, Universitetsparken 15, Copenhagen, Denmark University of Copenhagen Copenhagen Denmark

**Keywords:** biology, distribution, horse stomach bot fly, identification, nomenclature, taxonomy

## Abstract

A taxonomic review of *Gasterophilus* is presented, with nine valid species, 51 synonyms and misspellings for the genus and the species, updated diagnoses, worldwide distributions, and a summary of biological information for all species. Identification keys for adults and eggs are elaborated, based on a series of new diagnostic features and supported by high resolution photographs for adults. The genus is shown to have its highest species richness in China and South Africa, with seven species recorded, followed by Mongolia, Senegal, and Ukraine, with six species recorded.

## Introduction

The oestrids or bot flies (Oestridae) are known as obligate parasites of mammals in their larval stage. They are often highly host specific, and the short-lived, non-feeding adult flies may show remarkable patterns of camouflage or mimicry (Zumpt 1965; Grunin 1965, 1966, 1969; Guimarães and Papavero 1999; Colwell et al. 2006). Species of *Gasterophilus* Leach (Diptera: Oestridae, Gasterophilinae) are commonly known as horse stomach bot flies (from Greek: *gaster* for stomach, -*philus* indicating love or fondness). They have adapted to a larval life in the alimentary tract of Equidae (Zumpt 1965; Grunin 1969; Colwell et al. 2006), and their presence can lead to serious injuries or even death of the host (Hall and Wall 1995; Sequeira et al. 2001; Colwell et al. 2006; Bezdekova et al. 2007; Getachew et al. 2012). Because of their great veterinary importance, *Gasterophilus* species have received considerable attention since the early 1800s (Clark 1815; Dove 1918; Patton 1937; Zumpt and Paterson 1953; James 1974; Otranto et al. 2005a, b; Colwell et al. 2006, 2007; Zhang et al. 2016; Liu et al. 2016; Huang et al. 2017; Li et al. 2018). A total of more than 40 species-group names have been proposed for what is here recognized as nine valid species because of extensive intraspecific variation (Zumpt 1965; Grunin 1969; Pont 1980; Soós and Minář 1986; Cogley 1991a), and a series of misidentifications can be ascribed to their similar larval morphology (Colwell et al. 2007; Li et al. 2018). Zumpt (1965) and Grunin (1969) provided the basis of *Gasterophilus* taxonomy, and further taxonomic studies have been successively published, such as the recognition of *G. lativentris* (Brauer) as a synonym of *G. pecorum* (Fabricius) (Cogley 1991b) and the resurrection of *G. flavipes* (Olivier) [from synonymy with *G. haemorrhoidalis* (Linnaeus)] as a valid species (Li et al. 2019). Consequently, an update of the taxonomy, biology and distribution of *Gasterophilus* species was in demand.

*Gasterophilus* species were restricted to the Palaearctic and Afrotropical Regions, along with their equid hosts (Zumpt 1965; Leite et al. 1999), before becoming near cosmopolitan due to the association of several species with domestic hosts (Brauer 1863; Dove 1918; Zumpt 1965; Grunin 1969; Pont 1973; James 1974; Soós and Minář 1986; Wood 1987; Xue and Wang 1996; Colwell et al. 2006). Nonetheless, *G. meridionalis* (Pillers & Evans) and *G. ternicinctus* Gedoelst appear to be endemic to the Afrotropics, apparently exclusively associated with Burchell’s zebra (*Equus quagga burchellii* Gray) (Zumpt 1965); and *G. nigricornis* (Loew) is only recorded from eastern Europe and Central Asia in the Palaearctic Region (Zumpt 1965; Grunin 1969; Soós and Minář 1986; Xue and Wang 1996; Huang et al. 2017; Li et al. 2018). Records of *G. meridionalis* larvae in domestic horses from the Palaearctic Region (i.e., Iran, Italy, and Turkey) (Özdal et al. 2010; Mashayekhi and Ashtari 2013; Pilo et al. 2015) are suspected to be misidentifications (Li et al. 2018).

The life history of *Gasterophilus* species has been extensively investigated (Clark 1815; Dove 1918; Hadwen and Cameron 1918; Zumpt 1965; Grunin 1969; Catts 1979; Cogley and Cogley 2000; Anderson 2006; Colwell et al. 2006). The adults are known to live only 3–5 days, hovering around the host for ovipositing or gathering at hilltop aggregation sites for mating (Catts 1979). Females lay eggs directly on the host, attaching their eggs to the hairs of the lips, chin, cheeks, or forelegs, depending on the species (Dove 1918; Hadwen and Cameron 1918; Anderson 2006; Colwell et al. 2006; Wood 2006). One exception is *G. pecorum*, which attaches eggs to the tips of grass blades (Zumpt 1965; Grunin 1969). Larvae hatch spontaneously within 5–8 days, or when they are stimulated by moisture and friction associated with host licking, feeding or grooming. First instar larvae quickly penetrate into the host around the hatching site and migrate subcutaneously to the hosts’ mouth except for *G. nasalis*, which migrates on the mucosal surface to reach the inter-dental spaces (Zumpt 1965; Anderson 2006; Colwell et al. 2006). Each species of *Gasterophilus* has a specific site of penetration of the skin and route of migration to the stomach or intestine, where the second and third instar larval development is completed (Cogley et al. 1982; Colwell et al. 2006). It takes about 11 months for the larva to develop, with the third instar taking around 9–10 months in temperate climates. Mature larvae will be excreted with the feces and pupate in the soil (Zumpt 1965; Grunin 1969). The adults eclose after about 2–5 weeks and mate very soon after (Zumpt 1965; Anderson 2006).

Here, we take the opportunity to present an updated catalogue of all nine *Gasterophilus* species, including revised keys for eggs and adults, and updated diagnoses, host data, distributions, and original as well as major secondary literature for each species. This will be a help for entomologists, veterinarians, and other researchers with an interest in *Gasterophilus* to familiarize themselves more rapidly and more confidently in the taxonomy, biology, distribution, and literature on this group.

## Materials and methods

### Specimens

Label data provided under ‘Material examined’ are given in a standardized notation, with country names in capital letters and Chinese provinces in bold. Specimens studied or otherwise referred to are deposited in the following institutions:

**IOZ** Institute of Zoology, Chinese Academy of Sciences, Beijing, China

**KZNM** KwaZulu-Natal Museum, Pietermaritzburg, South Africa

**MBFU** Beijing Forestry University, Beijing, China

**MNHN** Museum national d’Histoire naturelle, Paris, France

**NHMUK** Natural History Museum, London, United Kingdom

**NHMD** Natural History Museum of Denmark, University of Copenhagen, Denmark

**NHMW** Naturhistorisches Museum Wien, Austria

**ZIN** Zoological Institute, Russian Academy of Sciences, St. Petersburg, Russia

### Imaging and terminology

A Visionary Digital Imaging System, with a Canon EOS 7D camera (Canon, Inc., Tokyo, Japan) was used to take series of photographs at the Natural History Museum of Denmark. Superimposed photographs were stacked using the Zerene Stacker software and composed using Adobe Photoshop CS6 (Adobe Systems, Inc., San Jose, CA, U.S.A.) on a Windows 10 platform.

Photographs are provided for *G. intestinalis* (De Geer), *G. meridionalis*, *G. nasalis* (Linnaeus), *G. nigricornis*, *G. ternicinctus*, and *G. pecorum*. High resolution photographs of *G. flavipes*, *G. haemorrhoidalis* and *G. inermis* (Brauer) were recently provided by Li et al. (2019).

Morphological terminology follows Cumming and Wood (2009) for adults and Ferrar (1987) for eggs.

### Distribution

A worldwide species diversity map was produced using the non-commercial version of StatPlanet (StatSilk 2018).

### Format of catalog

Regional catalogues (Pont 1973, 1980; Soós and Minář 1986) are followed with regard to synonyms as the valid names for species of *Gasterophilus* are accepted throughout current literature and the synonymies appear stable. All original proposals of available and unavailable names and first occurrences of misspellings were checked and updated for information on type locality. Generic synonyms are given with author, year: page, type species and mode of designation. The most important taxonomic, morphological, biological, distributional and evolutionary studies of *Gasterophilus* are selected and listed chronologically.

Valid species are treated in alphabetic order, with the valid name given in bold followed by a list of all synonyms in their original generic combination with author, year and page plus type locality given in modern English (with an original quotation where considered relevant, e.g., France, Pyrenees, “Dans les Pyrénées”). Precise localities provided by early authors are cited as well [e.g. Democratic Republic of the Congo (as “Zaire”), 11.5 km W of Luapula river (as “6 milles W. du Luapula”)]. Synonyms are listed chronologically for each species, followed by all published misspellings known to us. Most important references about taxonomic, morphological, biological, distributional and evolutionary studies of species in *Gasterophilus* are selected and listed chronologically.

Host records and distribution are given based on information from specimens examined for the present study (directly or from photos) and data from Brauer (1863), Zumpt (1965), Guimarães (1967), Grunin (1969), Pont (1973), James (1974), Kaboret et al. (1986), Soós and Minář (1986), Pearse et al. (1989), Pandey et al. (1992), Escartin and Bautista (1993), Xue and Wang (1996), Güiris et al. (2010), Özdal et al. (2010), Tavassoli and Bakht (2012), Mashayekhi and Ashtari (2013), Pape (2013), Ganjali and Keighobadi (2016), Huang et al. (2016), Hoseini et al. (2017), Muller and Ranwashe (2017), Tähtinen and Lahti (2017), van Noort and Ranwashe (2017). Host data is listed alphabetically, with both common name and scientific name. Distribution is given with countries listed alphabetically in their respective biogeographical regions, i.e., Afrotropical, Australasian, Nearctic, Neotropical, Palaearctic and Oriental Regions with boundaries as applied in Pape (1996). Further information, like Provinces or States, were given for countries with large continental area (i.e. Argentina, Australia, Brazil, Canada, Chile, China, United States of America), if applicable. Large islands (i.e., Corsica, Sardinia and Sicily) are listed together with their mainland countries. Non-vouchered literature records of *G. flavipes* obtained from Li et al. (2019) were retained with a question mark.

Biological information provided for eggs, larvae and adults is summarized and presented in Table 1.

**Table 1.** Natural history of *Gasterophilus* species.

Species	Embryonic development /days	Hatching strategy	First instar development	Second and third instar development	Pupal period /days	Host							
***G. flavipes***	NA	NA	NA	NA	NA	· Domestic donkey (*Equus africanus asinus* Linnaeus) [speculated by Brauer (1863) without evidence].	NA	NA	NA	NA	NA	NA	Brauer 1863; Li et al. 2019
***G. haemorrhoidalis***	2	Stimulated by moisture from licking or feeding of hosts.	· Penetrate epidermis of the lips of hosts and migrate into mouth.	· Second instar move to stomach and duodenum;	15–26	· Burchell’s zebra (*E. quagga burchellii* Gray);	50–200	Around host	Hairs along the edge of the lips	One egg per hair	‘Hit-and-flee’: female in full flight swiftly collides with a host and rapidly deposits an egg and then flies away before repeating the process	1–7	Dove 1918; Zumpt 1965; Colwell et al. 2006; Anderson 2006; Huang et al. 2016
				· Third instar larvae become detached after some time and then pass to the rectum and re-attach themselves.		· Domestic horse (*E. ferus caballus* Linnaeus)							
						· Domestic donkey;							
						· Mongolian wild ass (*E. hemionus hemionus* Pallas);							
						· Mountain zebra [*E. zebra* Linnaeus];							
						× Wild horse (*E. przewalskii* Poliakov)							
***G. inermis***	NA	Spontaneous.	· Penetrate skin of hosts at hatching site;	· Second and third instar larvae found in the rectum.	21–26	· Burchell’s zebra;	320–360	× Topographic landmark (Tops of hilltop shrubs/trees	Base of the hairs on cheeks	One egg per hair	Hit-and-flee	21–26	Zumpt 1965; Colwell et al. 2006; Anderson 2006; Huang et al. 2016
			· Migrate firstly under epidermis to the corner of mouth and then under the mucous membrane inside cheek.			· Domestic horse;							
						· Mongolian wild ass;							
						· Wild horse.							
***G. intestinalis***	5	Stimulated by moisture and friction supplied by rubbing and licking of hosts.	· Penetrate hosts’ dorsal mucosa of tongue;	· Young second instar larvae attach to the pharynx and the sides of the epiglottis, and then pass to the stomach;	22–28	· Domestic donkey;	400–1000	× Around host;	Distal half of the hairs on forelegs and chest	Often several eggs found on one hair	Female hovers slowly in one spot and quickly deposits several eggs before flying to another position or to another host	7–21	Dove 1918; Zumpt 1965; Catts 1979; Cogley et al. 1982; Colwell et al. 2006; Huang et al. 2016
			· Burrow from the anterior to posterior end; the migration route is almost parallel to the right or left lateral margin of tongue.	· Third instar larvae are generally found clustered near the boundary of the nonglandular and glandular epithelia.		· Domestic horse;		× Topographic landmark (hilltop, top of shrubs/trees					
						· Mongolian wild ass;							
						· Wild horse.							
***G. meridionalis***	NA	NA	NA	· Attached to stomach mucosa.	28–31	· Burchell’s zebra.	NA	NA	NA	NA	NA	NA	Zumpt 1965
***G. nasalis***	5–10	Spontaneous.	· Migrate on surface to inter-dental spaces of hosts.	· Moult to second instar at inter-dental sites;	16–24	· Burchell’s zebra;	300–500	Around host	Hairs under chin	Usually only one egg per hair, but occasionally five have been counted	Hit-and-flee, and an undisturbed female may deposit up to 20 eggs without leaving the host	1–12	Dove 1918; Zumpt 1965; Colwell et al. 2006; Anderson 2006; Huang et al. 2016
				· Migrate to duodenum and attach near pylorus.		· Domestic donkey;							
						· Domestic horse;							
						· Mongolian wild ass;							
						· Wild horse.							
***G. nigricornis***	3–9	Spontaneous.	· Penetrate hosts and migrate firstly under epidermis to the corner of mouth and then under the mucous membrane inside the cheek.	· Molt to the second stage in the central part of the cheek;	31–34	· Domestic donkey;	350–350	NA	Base of the hairs on cheek or neck	One egg per hair	Hit-and-flee	NA	Zumpt 1965; Colwell et al. 2006; Anderson 2006; Huang et al. 2016
				· Migrate to duodenum, attach to mucosa and become encysted;		· Domestic horse;							
				· Third instar larvae leave the cyst and become attached superficially to the mucous membrane.		· Mongolian wild ass;							
						· Wild horse.							
***G. pecorum***	5–8	Stimulated by moisture and friction supplied by hosts’ ingestion.	· Penetrate mouth mucosa of hosts;	· Molt to second and third instar at oral site;	12–21	· Burchell’s zebra;	1300–2600	Around host	Off host, mainly on tip of grass blades, and also on plant stems	In rows (groups of 10–15 eggs/batch	Female continuously lays several eggs in one spot before flying to another position	1–4	Zumpt 1965; Colwell et al. 2006; Hoseini et al. 2017; Huang et al. 2016
			· Migrate to the soft palate and at the root of the tongue, occasionally the pharynx and oesophagus.	· Third instars migrate to stomach and attach to mucosa.		· Domestic donkey;							
						· Domestic horse;							
						· Mongolian wild ass;							
						· Persian onager (*E. hemionus onager* Boddaert);							
						· Wild horse.							
***G. ternicinctus***	NA	NA	· NA	· Second and third instar larvae found in stomach.	20–27	× Burchell’s zebra.	NA	NA	NA	NA	NA	NA	Zumpt 1965

The generic diagnosis is provided for adults, eggs and larvae, while species diagnoses are provided only for adults. Keys are modified from already existing keys and updated with more diagnostic characters for both adults and eggs. Comprehensive identification keys to first instar larvae were published by Grunin (1969) and Zumpt (1965), and to third instar larvae by Li et al. (2018).

## Catalogue

### Genus Gasterophilus

Taxon classificationAnimaliaDipteraOestridae

14006216-1A40-5234-AA00-CCECF5EB7128

Figs 1 , 2 , 3 , 4 , 5 , 6 , 7 , 8 , 9 , 10 , 11 , 12 , 13 , 14 , 15 , 16 , 17 , 18 , 19 ; Table 1

Gasterophilus Leach, 1817: 2. Type species: Oestrus equi Clark, 1797 [= Oestrus intestinalis De Geer, 1776], by subsequent designation of Curtis (1826: 146).
Gastrus Meigen, 1824: 174. Type species: Oestrus intestinalis De Geer, 1776, by subsequent designation of Coquillett (1910: 546).
Gastrophilus Agassiz, 1846: 160. Unjustified emendation of Gasterophilus Leach, 1817. Type species: Oestrus equi Clark, 1797 [= Oestrus intestinalis De Geer, 1776] (automatic).
Enteromyza Rondani, 1857: 20. Unnecessary new replacement name for Gastrus Meigen, 1824 and Gasterophilus Leach, 1817. Type species: Oestrus equi Clark, 1797 [= Oestrus intestinalis De Geer, 1776] (automatic).
Rhinogastrophilus Townsend, 1918: 152. Type species: Oestrus nasalis Linnaeus, 1758, by original designation.
Enteromyia Enderlein, 1934: 425. Type species: Oestrus haemorrhoidalis Linnaeus, 1758, by original designation.
Stomachobia Enderlein, 1934: 425. Type species: Oestrus pecorum Fabricius, 1794, by original designation.
Haemorrhoestrus Townsend, 1934: 406. Type species: Oestrus haemorrhoidalis Linnaeus, 1758, by original designation.
Progastrophilus Townsend, 1934: 406. Type species: Oestrus pecorum Fabricius, 1794, by original designation.

#### Selected references.

Brauer (1863: 53); Zumpt (1965: 111); Grunin (1969: 21); Pont (1973: 698); James (1974: 92); Kettle (1974); Papavero (1977: 19); Wood (1987: 1148); Soós and Minář (1986: 238); Xue and Wang (1996: 2209); Pape (2001); Pape et al. (2017); Otranto et al. (2005); Colwell et al. (2006: 5); Colwell et al. (2007); Felix et al. (2007); Zhang et al. (2012); Huang et al. (2016); Zhang et al. (2016); Li et al. (2018, 2019); Yan et al. (2019).

#### Diagnosis.

**Figure 1.**
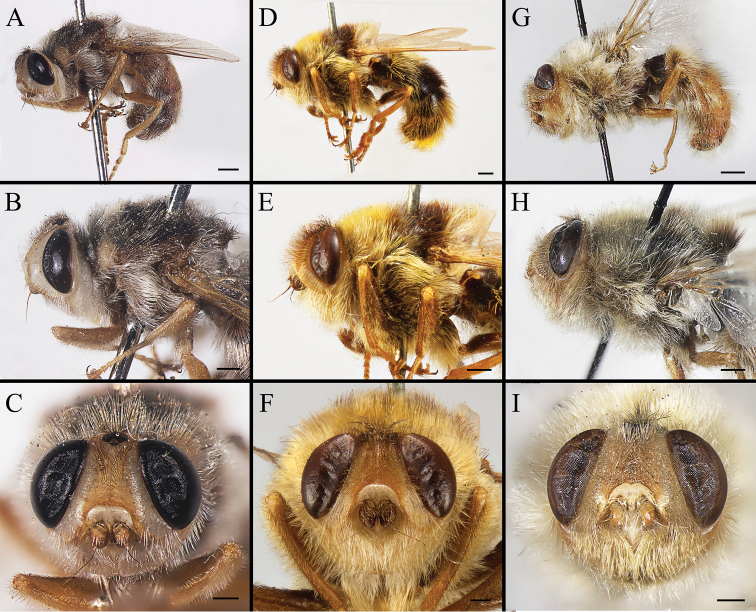
Left lateral view of habitus (**A, D, G**), head and thorax (**B, E, H**), and head in frontal view (**C, F, I**) of male *Gasterophilus* species, modified from Li et al. (2019)**A–C***G. flavipes* (Olivier); Morocco (in IOZ) **D–F***G. haemorrhoidalis* (Linnaeus); China (in MBFU) **G–I***G. inermis* (Brauer); Germany (in NHMD). Scale bars: 1 mm (**A, D, G**); 0.5 mm (**B, C, E, F, H, I**).

**Figure 2.**
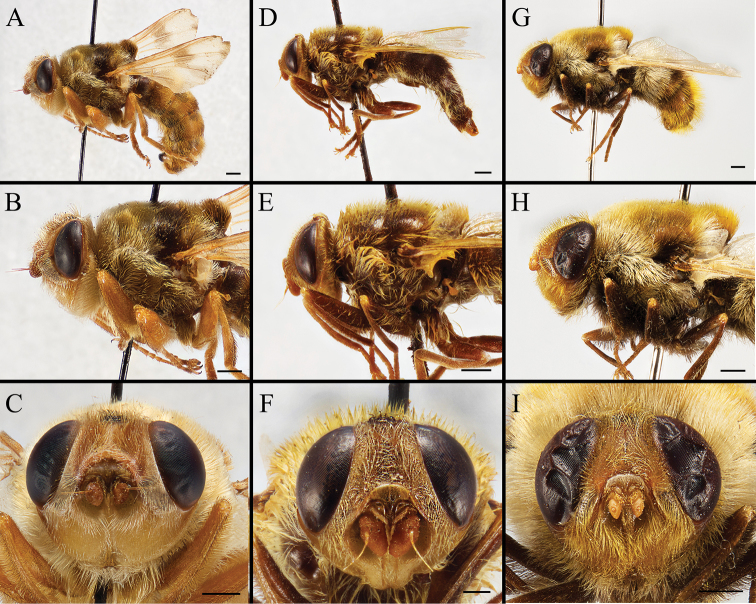
Left lateral view of habitus (**A, D, G**), head and thorax (**B, E, H**), and head in frontal view (**C, F, I**) of *Gasterophilus* species **A–C** Male *G. intestinalis* (De Geer) China (in MBFU) **D–F** Female *G. meridionalis* (Pillers & Evans); South Africa (in KZNM) **G–I** Male *G. nasalis* (Linnaeus) China (in MBFU). Scale bars: 1 mm (**A–E, I**); 0.5 mm (**F**).

**Figure 3.**
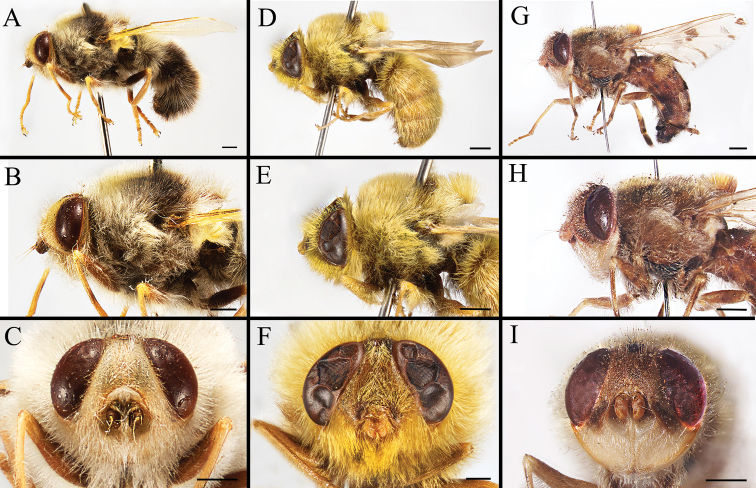
Left lateral view of habitus (**A, D, G**), head and thorax (**B, E, H**), and head in frontal view (**C, F, I**) of male *Gasterophilus* species **A–C***G. nigricornis* (Loew); China (in MBFU) **D–F***G. pecorum* (Fabricius); China (in MBFU) **G–I***G. ternicinctus* Gedoelst; South Africa (in MBFU). Scale bars: 1 mm (**A–I**).

**Figure 4.**
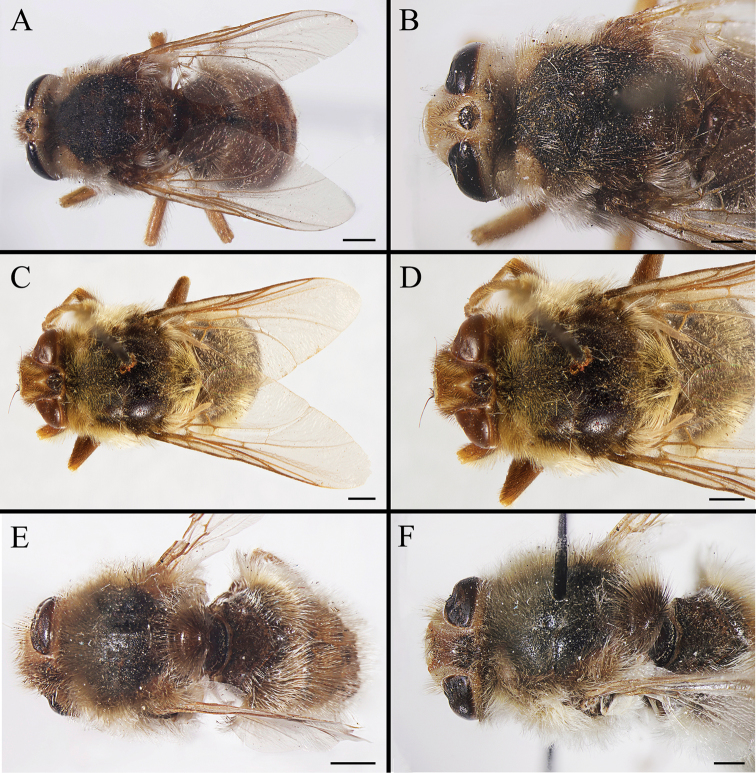
Dorsal view of habitus (**A, C, E**) and head and thorax (**B, D, F**) of male *Gasterophilus* species, modified from Li et al. (2019)**A, B***G. flavipes* (Olivier) **C, D***G. haemorrhoidalis* (Linnaeus) **E, F***G. inermis* (Brauer). Scale bars: 1 mm (**A, C–D, E**); 0.5 mm (**B, F**).

**Figure 5.**
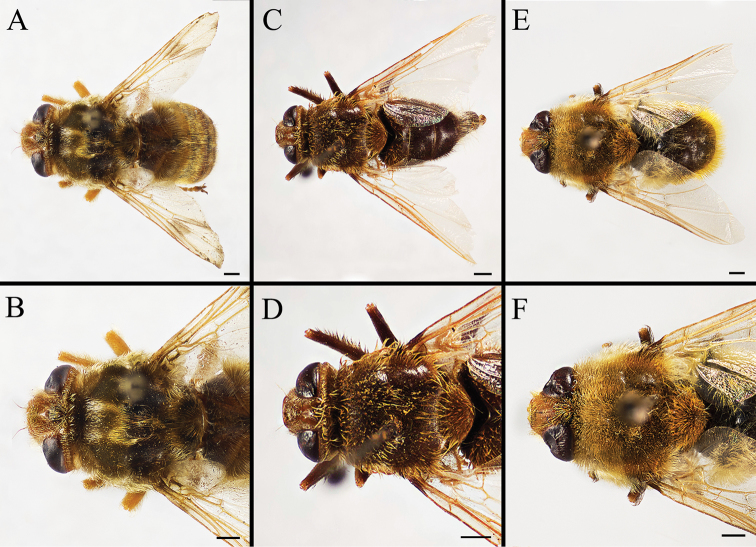
Dorsal view of habitus (**A, C, E**) and head and and thorax (**B, D, F**) of male *Gasterophilus* species **A, B** Male *G. intestinalis* (De Geer) **C, D** Female *G. meridionalis* (Pillers & Evans) **E, F** Male *G. nasalis* (Linnaeus). Scale bars: 1 mm (**A–F**).

**Figure 6.**
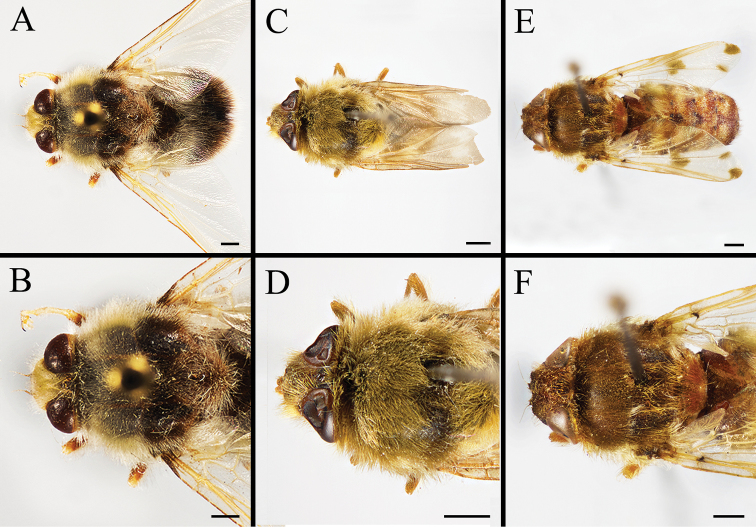
Dorsal view of habitus (**A, C, E**) and head and thorax (**B, D, F**) of male *Gasterophilus* species **A, B***G. nigricornis* (Loew) **C, D***G. pecorum* (Fabricius) **E, F***G. ternicinctus* Gedoelst. Scale bars: 1 mm (**A–F**).

**Figure 7.**
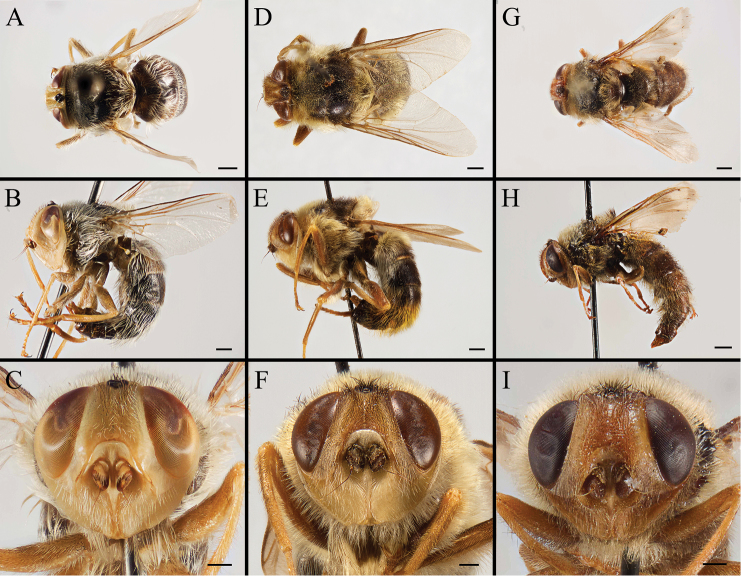
Dorsal view (**A, D, G**) and left lateral view (**B, E, H**) of habitus, and head in frontal view (**C, F, I**) of female *Gasterophilus* species, modified from Li et al. (2019)**A–C***G. flavipes* (Olivier); China (in MBFU) **D–F***G. haemorrhoidalis* (Linnaeus); China (in MBFU) **G–I***G. inermis* (Brauer); Germany (in NHMD). Scale bars: 1 mm (**A, B, D, E, G, H**); 0.5 mm (**C, F, I**).

**Figure 8.**
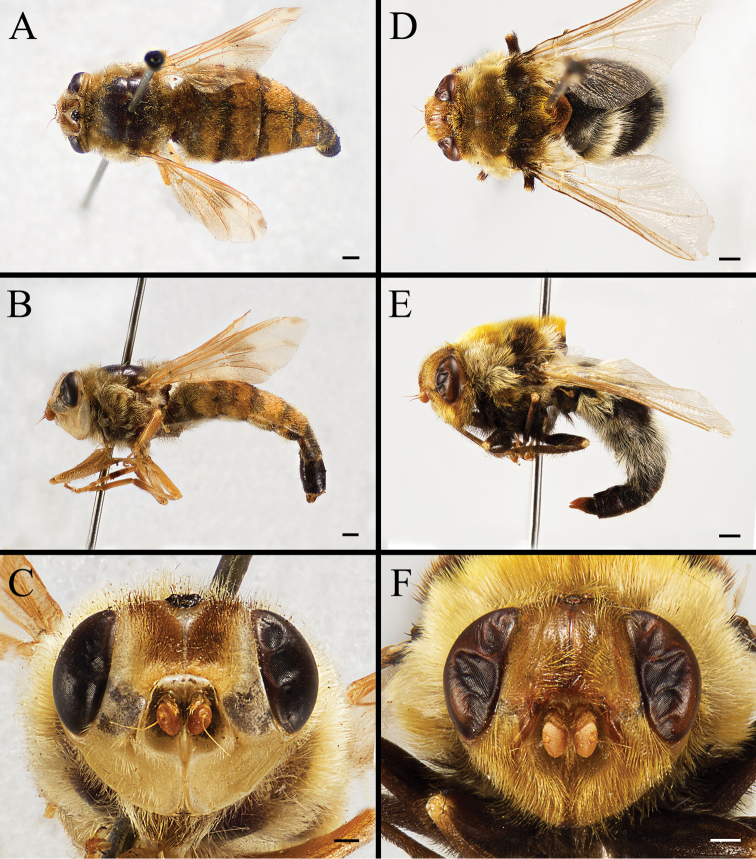
Dorsal view (**A, D, G**) and left lateral view (**B, E, H**) of habitus, and head in frontal view (**C, F, I**) of female *Gasterophilus* species **A–C***G. intestinalis* (De Geer); China (in MBFU) **D–F***G. nasalis* (Linnaeus); China (in MBFU). Scale bars: 1 mm (**A, B, D, E**); 0.5 mm (**C, F**).

**Figure 9.**
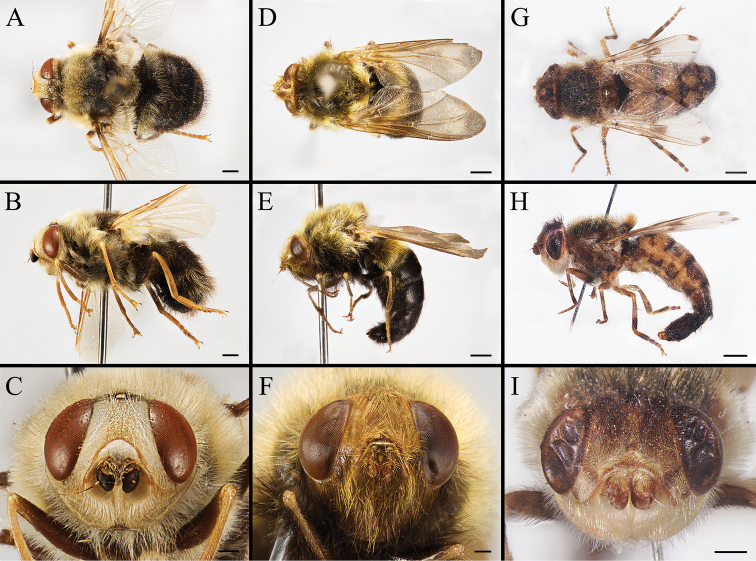
Dorsal view (**A, D, G**) and left lateral view (**B, E, H**) of habitus, and head in frontal view (**C, F, I**) of female *Gasterophilus* species **A–C***G. nigricornis* (Loew); China (in MBFU) **D–F***G. pecorum* (Fabricius); China (in MBFU) **G–I***G. ternicinctus* Gedoelst; Kenya (in NHM). Scale bars: 1 mm (**A, B, D–E, G, H**); 0.5 mm (**C, F, I**).

**Figure 10.**
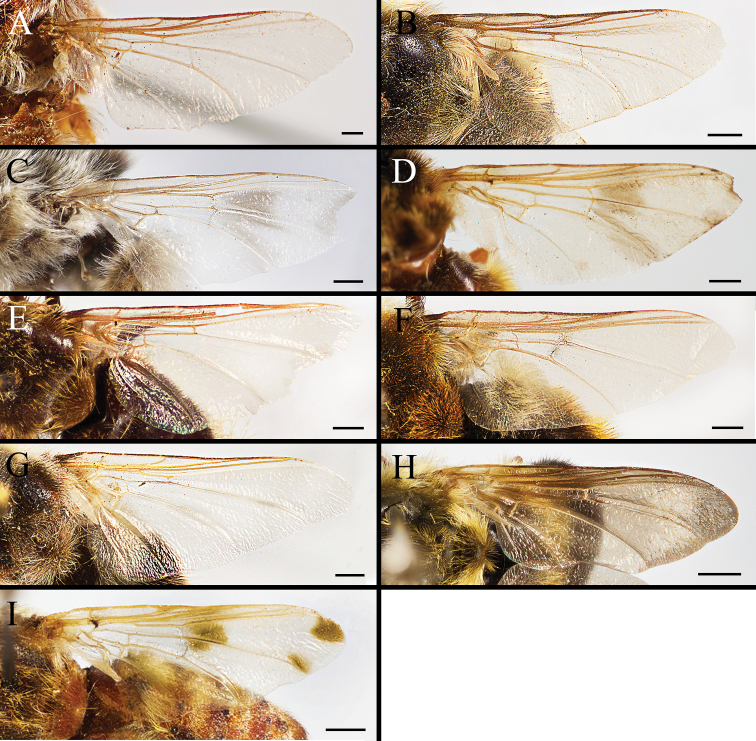
Wing of *Gasterophilus* species, with **A–C** modified from Li et al. (2019)**A***G. flavipes* (Olivier) **B***G. haemorrhoidalis* (Linnaeus) **C***G. inermis* (Brauer) **D***G. intestinalis***E***G. meridionalis* (Pillers & Evans) **F***G. nasalis* (Linnaeus) **G***G. nigricornis* (Loew) **H***G. pecorum* (Fabricius) **I***G. ternicinctus* Gedoelst. Scale bars: 0.5 mm (**A–C**); 1 mm (**D–I**).

**Figure 11.**
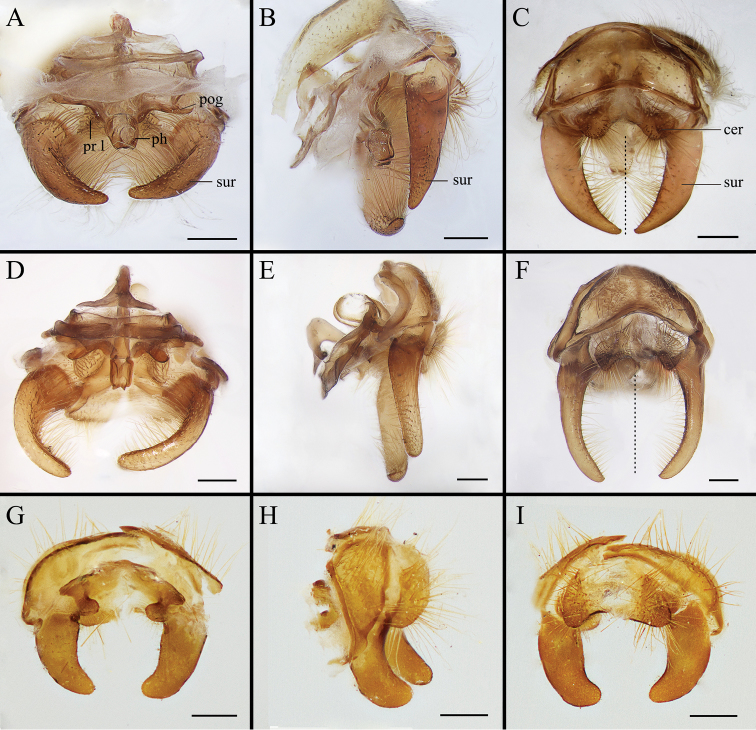
Anterior (**A, D, G**), left lateral (**B, E, H**) and posterior view (**C, F, I**) of male terminalia of *Gasterophilus* species, modified from Li et al. (2019)**A–C***G. flavipes* (Olivier) **D–F***G. haemorrhoidalis* (Linnaeus) **G–I***G. inermis* (Brauer). Scale bars: 0.5 mm (**A–I**). The dotted line in **C** and **F** indicates the sagittal plane. Abbreviations: cer, cercus; ph, phallus; pog, postgonite; pr l, processi longi; sur, sustylus.

**Figure 12.**
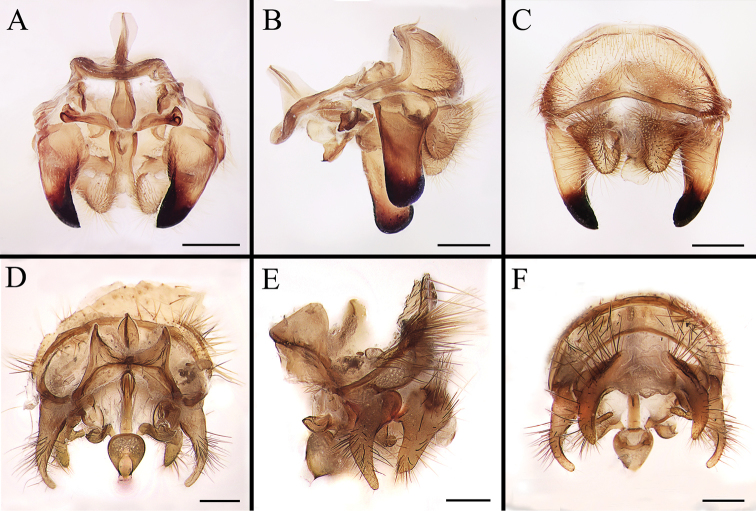
Dorsal (**A, D**), left lateral (**B, E**) and posterior (**C, F**) view of male genitalia of *Gasterophilus* species **A–C***G. intestinalis* (De Geer) **D–F***G. nasalis* (Linnaeus). Scale bars: 0.5 mm (**A–F**).

**Figure 13.**
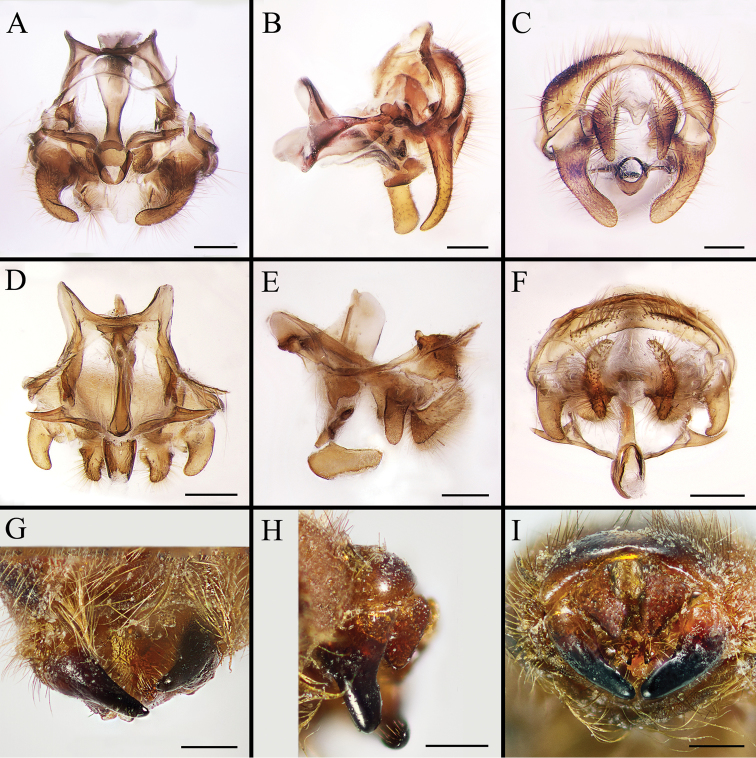
Dorsal (**A, D, G**), left lateral (**B, E, H**) and posterior (**C, F, I**) view of male genitalia of *Gasterophilus* species **A–C***G. nigricornis* (Loew) **D–F***G. pecorum* (Fabricius) **G–I***G. ternicinctus* Gedoelst. Scale bars: 0.5 mm (**A–I**).

**Figure 14.**
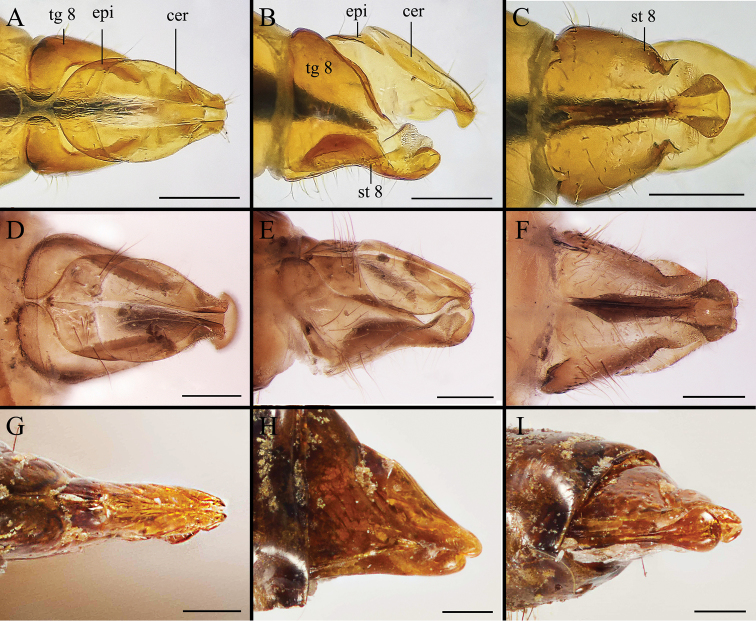
Dorsal (**A, D, G**), left lateral (**B, E, H**) and ventral (**C, F, I**) view of female genitalia of *Gasterophilus* species, modified from Li et al. (2019)**A–C***G. flavipes* (Olivier) **D–F***G. haemorrhoidalis* (Linnaeus) **G–I***G. inermis* (Brauer). Scale bars: 0.5 mm (**A–C**); 1 mm (**D–I**). Abbreviations: cer, cercus; epi, epiproct; sg 7, segment 7; sl, stalk-like pedicel; st 8, sternite 8; tg 8, tergite 8.

**Figure 15.**
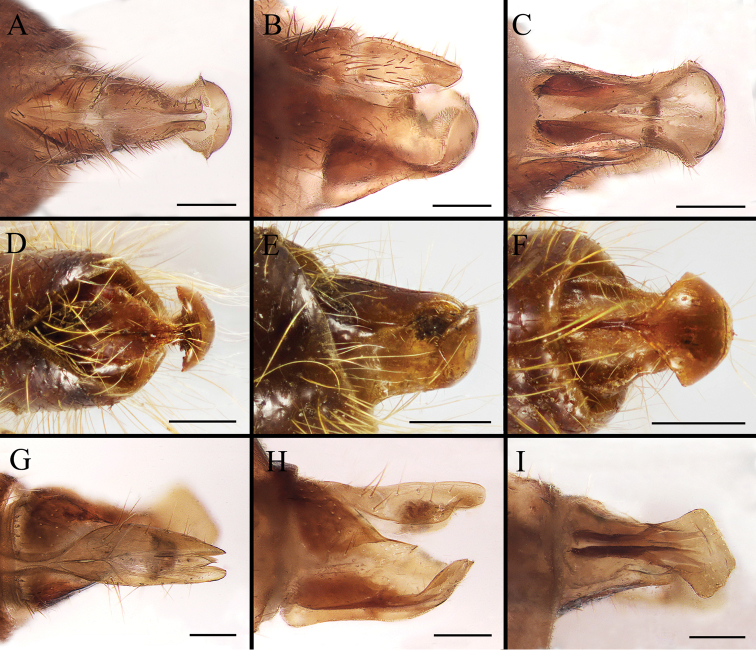
Dorsal (**A, D, G**), left lateral (**B, E, H**) and ventral (**C, F, I**) view of female genitalia of *Gasterophilus* species **A–C***G. intestinalis* (De Geer) **D–F***G. meridionalis* (Pillers & Evans) **G–I***G. nasalis* (Linnaeus). Scale bars: 0.5 mm (**A–I**).

**Figure 16.**
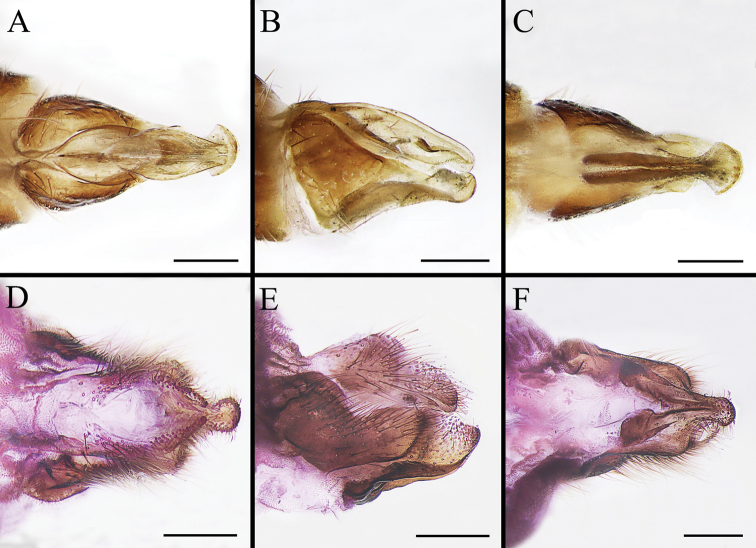
Dorsal (**A, D**), left lateral (**B, E**) and ventral (**C, F**) view of female genitalia of *Gasterophilus* species **A–C***G. nigricornis* (Loew) **D–F***G. pecorum* (Fabricius). Scale bars: 0.5 mm (**A–F**).

**Figure 17.**
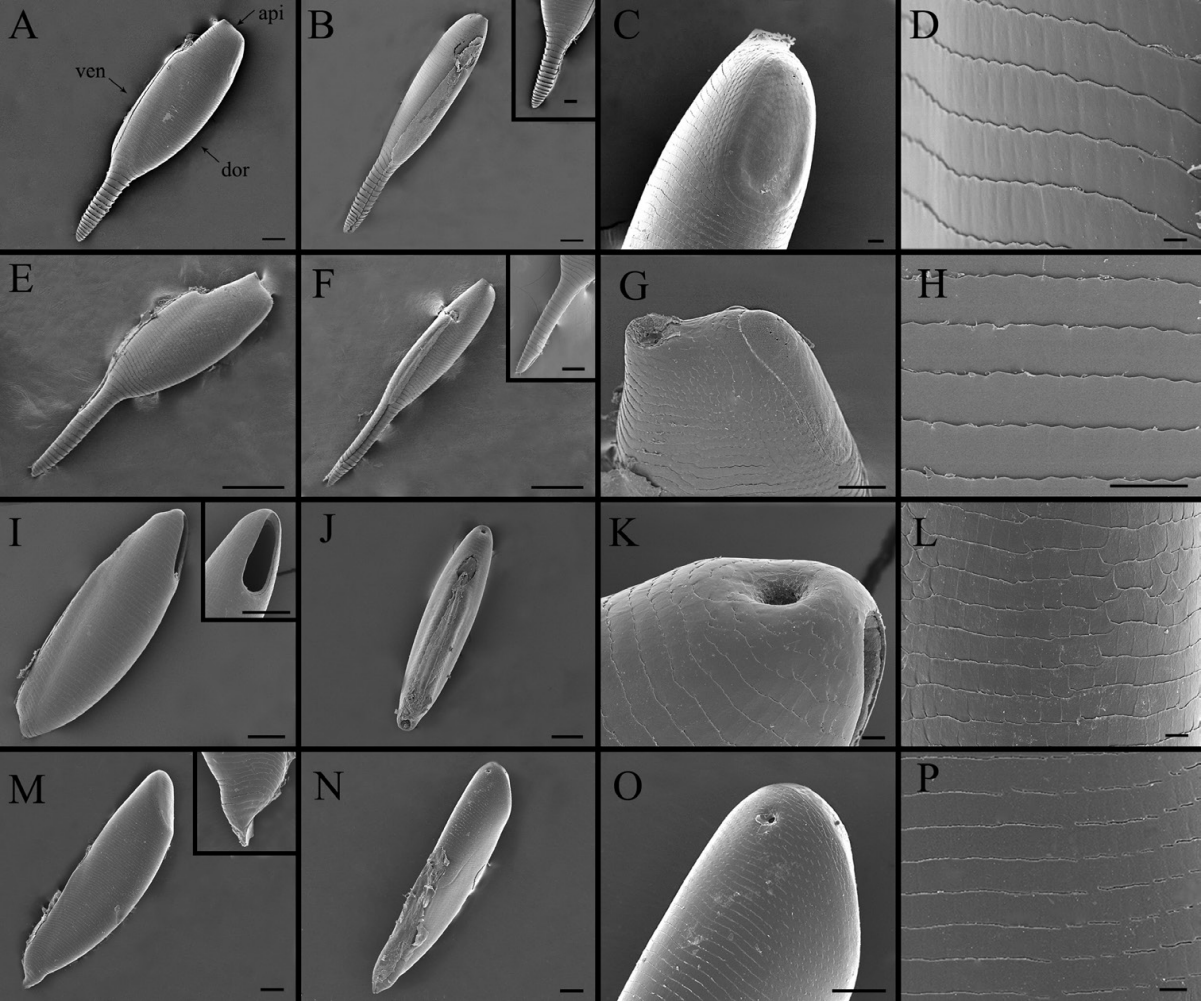
Right lateral (**A, E, I, M**) and ventral (**B, F, J, N**) view, micropyle (**C, G, K, O**) and ultrastructural details of plastron (**D, H, L, P**) of eggs in *Gasterophilus* species **A–D***G. flavipes* (Olivier) **E–H***G. haemorrhoidalis* (Linnaeus) **I–L***G. inermis* (Brauer) **M–P***G. intestinalis*. Abbreviations: api, apical; dor, dorsal; ven, ventral. Scale bars: 100 μm (**A, B, I–J, M, –N**), 50 μm (in box of **B**), 100 μm (in box of **I**); 20 μm (**C**); 5 μm (**D**); 250 μm (**E, F**), 20 μm (in box of **F**); 50 μm (**G, O**); 25 μm (**H**); 10 μm (**K, L, P**).

**Figure 18.**
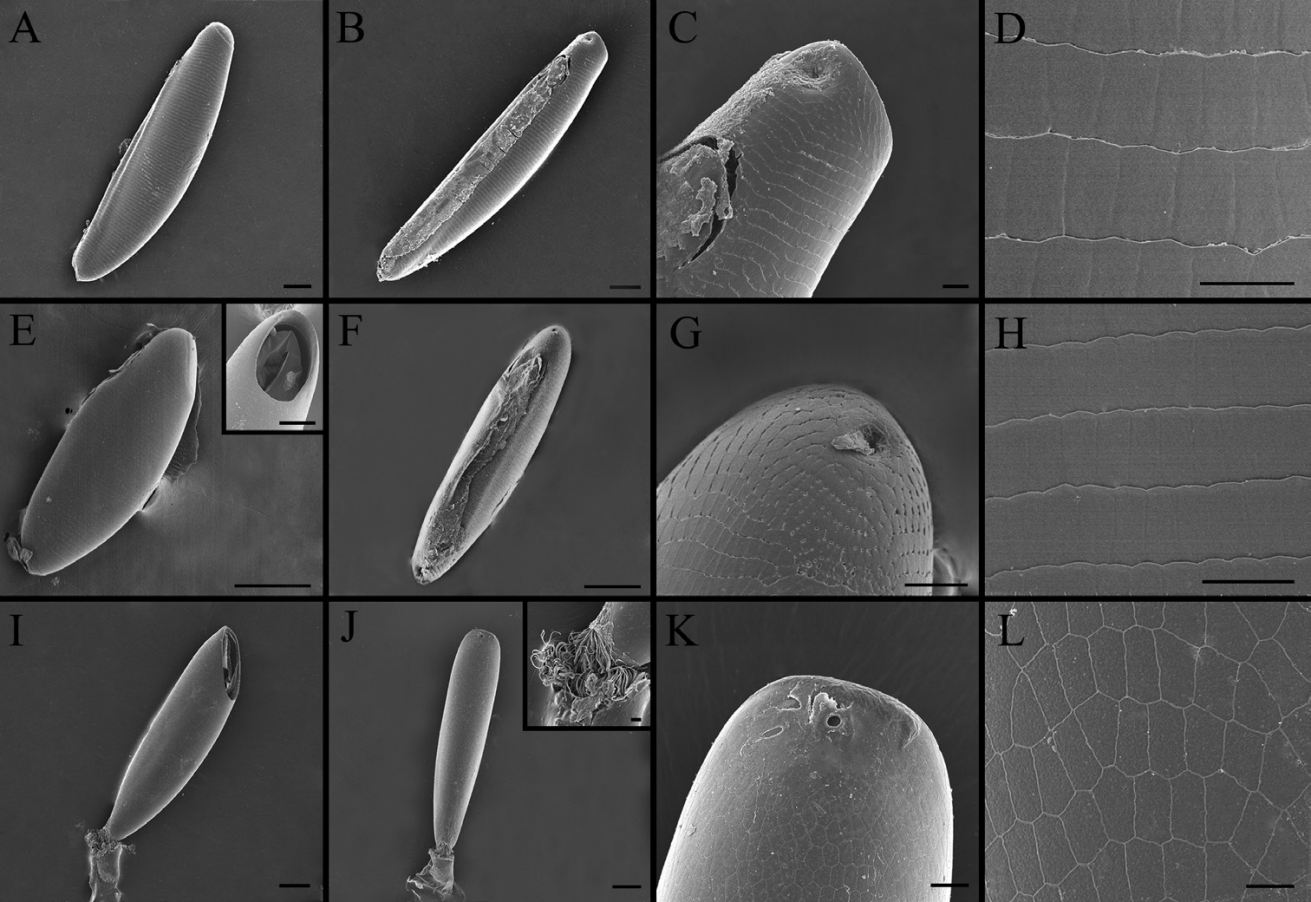
Right lateral (**A, E, I**) and ventral (**B, F, J**) view, micropyle (**C, G, K**) and ultrastructural details of plastron (**D, H, L**) of eggs in *Gasterophilus* species **A–D***G. nasalis* (Linnaeus) **E–H***G. nigricornis* (Loew) **I–L***G. pecorum* (Fabricius). Scale bars: 100 μm (**A, B, I–J**), 10 μm (in the box of **J**); 20 μm (**C, H, K**); 15 μm (**D**); 20 μm (**E**), 50 μm (in the box of **E**); 150 μm (**F**); 25 μm (**G**); 10 μm (**L**).

Body covered with dense, yellowish hair-like setae, variously interrupted by reddish-yellow or dark brown (or black) bands (Figs 1–10). Facial plate with a narrow median keel. Antennal arista long, slender, gradually tapered and slightly flattened, with short, sparse microtrichia (Figs 1C, F, I, 2C, F, I, 3C, F, I, 7C, F, I, 8C, F, I, 9C, F, I). Proboscis and palpus vestigial, visible as small, yellow or brown knobs. Thorax ground color mainly dark brown or black (Figs 4–6, 7A, D, G, 8A, D, G, 9A, D, G). Notopleuron weakly defined. Posterior spiracle open, with short, hair-like fringes, lappets oriented obliquely at an angle of about 45 degrees. Wing vein M almost straight, very slightly curved posteriorly; vein A_1_ + CuA_2_ extending to wing margin (Fig. 10). Upper and lower calypters yellowish, fringed with long, whitish, hair-like setae along the external margin. Abdomen ground color yellow, dark brown or black, sometimes with several irregular dark spots (Figs 1A, D, G, 2A, D, G, 3A, D, G, 4A, C, E, 5A, C, E, 6A, C, E, 7A, B, D, E, 8A, B, D, E, 9A, B, D, E, G, H). Male cercus (Figs 11–13) broadly connected to its counterpart by a membrane at the base, with a long or short free apex (Figs 11C, F, I, 12C, F, 13C, F, I); surstylus with a rounded or gradually tapered apex (Figs 11B, E, H; 12B, E; 13B, E, H); phallus short, dorsolateral processes of distiphallus reduced, epiphallus absent; pregonite tuberculous; postgonite falcate (Figs 11A, D, G, 12A, D, 13A, D, G); processi longi (remnants of sternite 10) setose, tubercular or elongated (Patton 1937, Grunin 1969). Female terminalia (Figs 14–16) gradually tapered, either short and straight (Fig. 9E) or long and curved forward (Figs 7B, E, H, 8B, E, 9B, H); segment 7 modified, fully sclerotized, tube-shaped, dorsally with a longitudinal suture, without separation of tergite and sternite 7; tergite 8 laterally expanded downwards; sternite 8 either with a longitudinal concavity in the middle and with a keel-shaped apex (Fig. 16F), or longitudinally ridged in the middle and with a scallop-shaped apex (Figs 14C, F, L, 15C, F, L, 16C); tergite 10 (epiproct) composed of two approximately triangular sclerites (Figs 14B, E, H, 15B, E, H, 16B, E); cercus long and narrow, narrowly connected to its counterpart by membrane and with a very short prolongation (Figs 14A, D, G, 15A, D, G, 16A, D). Eggs with an attachment organ, short and posteriorly located or elongated and situated ventrally (Figs 17–18). The larva with a bilobed, highly constricted pseudocephalon, three thoracic segments, seven abdominal segments, and the anal division divided into three subdivisions (Zumpt 1965; Grunin 1969; Li et al. 2018). The freshly hatched larva fusiform, anteriorly encircled with strong body spinose; posterior spiracles slightly or distinctly elongated, fully exposed, with two serrated margined slits (Zumpt 1965; Grunin 1969). The second and third instar larva sub-cylindrical, with mouth hooks posterolaterally curved and sharply pointed, and a pair of oral plates between mouth hooks; most of the body segments circled anteriorly by strong, posteriorly directed spines arranged in one, two or three rows (Zumpt 1965; Grunin 1969; Li et al. 2018). The third instar larva distinctively colored in red, yellow or green (Li et al. 2018).

#### Hosts.

Known exclusively from the genus *Equus* Linnaeus (Perissodactyla: Equidae). So far, no records have been made from the species *E. grevyi* Oustalet (Grévy’s zebra) and *E. kiang* Moorcroft (kiang or Tibetan wild ass).

#### Distribution and diversity.

**Figure 19.**
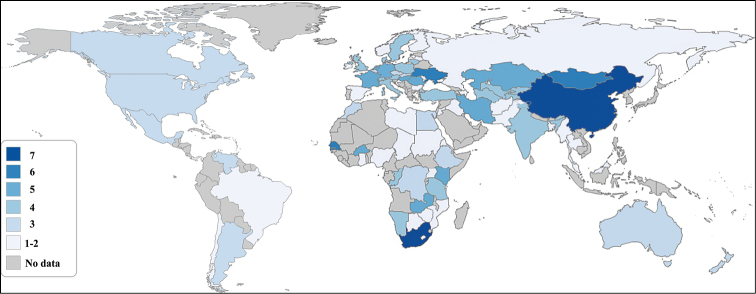
Species diversity map of all nine *Gasterophilus* species worldwide. Different colors represent the number of species recorded in a specific country. Interactive map showing the global distribution of all nine *Gasterophilus* species is available in Supplementary Information 1.

Native distribution matches that of the horse family, currently with highest diversity in China and South Africa, with 7 species recorded, followed by Mongolia, Senegal and Ukraine, with 6 species recorded (Fig. 19). Introduced with domestic hosts to most parts of the world.

##### Key to adults of *Gasterophilus* spp.

**Table:** 

1	Wing with darkened patches (Fig. 10C, D, H, I)	**2**
–	Wing entirely hyaline (Fig. 10A, B, E–G)	**5**
2	Wing patches sharply demarcated (Fig. 10I); hind tibia and tarsus distinctly flattened (to a lesser degree in female), tarsomeres 2–4 shortened, as broad as long or broader than long (Figs 3D, 9G, H)	***Gasterophilus ternicinctus***
–	Wing patches with ill-defined edges (Figs 10C, D, H); hind tibia and tarsus unmodified, tarsomeres 2–4 long and narrow, distinctly longer than broad	**3**
3	Antennal pedicel elongated, length/width ratio more than 0.8 (Figs 3F, 9F); facial plate setose; abdomen ground color yellow in male (Fig. 3D), mainly black in female (Fig. 9E); female terminalia short (Fig. 9E), abdominal sternite 8 with a keel-shaped apex (Fig. 16F)	***Gasterophilus pecorum***
–	Antennal pedicel short, length/width ratio less than 0.5; facial plate bare; abdomen ground color mainly yellow; female terminalia elongated, abdominal sternite 8 with a scallop-shaped apex (Figs 15C, F, I, 16C)	**4**
4	Hind trochanter ventrally with a spatulate process in male or a tubercle in female (Grunin 1969: fig. 95); male surstylus yellow, with a black apex (Fig. 12A–C); female abdominal segment 7 longer than broad (Fig. 8B)	***Gasterophilus intestinalis***
–	Hind trochanter without a process or tubercle; male surstylus entirely yellow (Grunin 1969: fig. 86); female abdomen abdominal segment 7 broader than long (Fig. 7H)	***Gasterophilus inermis***
5	Crossvein dm-cu present; antennal postpedicel yellow or brownish; meral setae unmodified	**6**
–	Crossvein dm-cu absent (Fig. 10G); antennal postpedicel red-brown to blackish (Figs 3C, 9C); meral setae with tip swollen	***Gasterophilus nigricornis***
6	Crossvein dm-cu distinct; antennal postpedicel globular	**7**
–	Crossvein dm-cu extremely faint (Fig. 10E); antennal postpedicel long-oval (Fig. 2F)	***Gasterophilus meridionalis***
7	Distance between crossveins r-m and dm-cu at least twice as long as r-m; male cercus short and broad, length/width ratio equal to or less than 1.0, surstylus much longer than cercus	**8**
–	Distance between crossveins r-m and dm-cu less than length of r-m (Fig. 10F); male cercus long and narrow, length/width ratio more than 3.0, surstylus and cercus of similar length (Fig. 12D–F)	***Gasterophilus nasalis***
8	Postsutural scutum with a light (yellowish), rectangular area near scutoscutellar suture (Fig. 4A); legs yellow; abdomen ground color yellow, covered with yellow setae (Figs 1A, 4A, 7A–B); male with surstylus gradually tapered proximally and distally, surstylar setae long, reaching the sagittal plane (Fig. 11A–C)	***Gasterophilus flavipes***
–	Postsutural scutum with ground color uniformly brown or black (Figs 4C, D, 7D); legs yellowish brown, with femora distinctly darkened; abdomen ground color dark brown or black, with reddish-yellow or orangish setae posteriorly (Figs 1D, 7E); male with surstylus abruptly tapered distally, surstylar setae short, reaching at most halfway to the sagittal plane (Fig. 11D–F)	***Gasterophilus haemorrhoidalis***

##### Key to eggs of *Gasterophilus* spp.

**Table:** 

1	Posteriorly with an elongated pedicel (a continuation of the broad chorionic flanges) (Fig. 17A, B, E, F)	**2**
–	Posteriorly with a very short pedicel or without a pedicel (Figs 17I, J, M, N, 18A, B, E, F, I, J)	**3**
2	Pedicel short and thick, with width/length ratio around 1/4 in lateral view, accounting for 1/3 of the total egg length (Fig. 16A–D)	***Gasterophilus flavipes***
–	Pedicel long and slender, with width/length ratio around 1/6 in lateral view, accounting for 2/5 of the total egg length (Fig. 17E–H)	***Gasterophilus haemorrhoidalis***
3	Chorion brownish black, posteriorly with a short attachment organ, accounting for 1/6 of egg length (Fig. 18I–L)	***Gasterophilus pecorum***
–	Chorion yellowish, ventrally with a long attachment organ, accounting for at least 1/2 of egg length	**4**
4	Egg gradually tapered, anterior half distinctly broader than posterior half (Fig. 17M–P)	***Gasterophilus intestinalis***
–	Egg fusiform, swollen in the middle, anteriorly and posteriorly tapered	**5**
5	Attachment organ around half the length of the egg (Cogley 1991b: fig. 8)	***Gasterophilus ternicinctus***
–	Attachment organ almost the same length as the egg	**6**
6	Operculum placed apically (parallel to the egg’s cross section) (Fig. 18A)	***Gasterophilus nasalis***
–	Operculum placed sub-apically (distinctly angled relative to the egg’s cross section) (Figs 17I, 18E)	**7**
7	Micropylar position apical (on top surface) (Cogley 1991b: fig. 9)	***Gasterophilus meridionalis***
–	Micropylar position sub-apical (on ventral surface)	**8**
8	Operculum length/width ratio about 2.0 (Fig. 18E)	***Gasterophilus nigricornis***
–	Operculum length/width ratio about 4.0 (Fig. 17I)	***Gasterophilus inermis***

### Gasterophilus flavipes

Taxon classificationAnimaliaDipteraOestridae

(Oliver, 1811)

5447CFF1-C822-5E98-A503-A8A807BCDEEC

Figs 1A–C , 4A, B , 7A–C , 10A , 11A–C , 14A–C , 17A–D ; Table 1

Oestrus flavipes Olivier, 1811: 467. Type locality: France, Pyrenees (“Dans les Pyrénées”).

#### Selected references.

Brauer (1863: 80); Patton (1937); Li et al. (2019).

#### Diagnosis.

Facial plate bare. Postsutural scutum of light color (yellowish), with rectangular area near scutoscutellar suture. Wing completely hyaline. Distance between crossveins r-m and dm-cu at least twice as long as r-m. Meron with unmodified setae. Legs yellow; hind tarsus with long, strong and dense setae ventrolaterally. Abdomen ground color yellow. Male cercus short and broad, length/width ratio equal or less than 1.0; surstylus yellow, gradually tapered proximally and distally, with a gradually tapered apex; surstylar setae long, reaching the sagittal plane; processi longi tubercular. Female sternite 8 longitudinally ridged in the middle and with a scallop-shaped apex.

#### Material examined.

CHINA – **Xinjiang Uyghur Autonomous Region** • 10♂♂, 14♀♀; Kalamaili, Qiaomuxibai water reservoir; 45°13.8'N, 89°3.0'E (DDM); 1000 m; 26 Jun 2017; Y.Q. Ge & W.Y. Pei leg.; MBFU • 1♂, 1♀; same data as for preceding; NHMD. – **Inner Mongolia** • 1♂; Chifeng; 1 Jul. 1960, collector unknown; IOZ • 1♂; same collection locality as for preceding; 3 Jul. 1960; collector unknown; IOZ. CYPRUS • 1♂; no further data; NHMUK. MOROCCO • 1♂; no further data; 1897; G. Buchet leg.; MNHN • 1♂; Haute Moulouya; 1918; Thullet leg.; MNHN •1♂; Moyen Atlas; Hun. 1949; L. Chopard leg.; MNHN • 1♀; LIBYA • Zuwarah; no further data; NHMUK • 1♂; SUDAN • Ed Dueim; 1937; collector unknown; NHMUK • 1♂; no further data; NHMUK.

#### Hosts.

Donkey (*E. africanus asinus* Linnaeus) [speculated by Brauer (1863) without evidence].

#### Distribution.

**Afrotropical** – Sudan. **Palaearctic** – China (Inner Mongolia, Xinjiang), Croatia?, Cyprus, Egypt?, France, Iran?, Kazakhstan?, Libya, Morocco, Spain?, Turkey?

### Gasterophilus haemorrhoidalis

Taxon classificationAnimaliaDipteraOestridae

(Linnaeus, 1758)

5F417E39-848C-5685-AFF6-D451114C5B59

Figs 1D–F , 4C, D , 7D–F , 10B , 11D–F , 14D–F , 17E–H ; Table 1

Oestrus haemorrhoidalis Linnaeus, 1758: 584 (as “hæmorrhoidalis”). Type locality: not given, probably Sweden, Germany, and France (through reference to *Fauna Svecica* and unspecified works by Johann Leonhard Frisch and Antoine Ferchault de Réaumur).
Oestrus salutiferus Clark, 1816: 3. Type locality: England.
Oestrus duodenalis Schwab, 1840: 35. Type locality: Europe. Proposed in synonymy with Oestrus salutiferus Clark, 1816, made available from subsequent use as a valid name for a taxon by Gistel (1848: 153).
Gastrophilus pallens Bigot, 1884: 4. Type locality: Sudan, Suakin (as “Suakim? Soudan oriental”).
Gasterophilus pseudohaemorrhoidalis Gedoelst, 1923: 272 (as “*pseudo-haemorrhoïdalis*”). Type locality: Eritrea, Asmara (as “Erythree: Asmara”); Republic of the Congo, Katanga Province, Biano (as “Katanga: Biano”) and Zambia (as “Zambi”).
Oestrus hemorrhoidalis Clark, 1815: 71; incorrect subsequent spelling of haemorrhoidalis Linnaeus, 1758.
Oestrus haemorrhoidales Clark, 1816: [1]; incorrect subsequent spelling of haemorrhoidalis Linnaeus, 1758.
Oestrus hemorroidalis Guérin-Méneville, 1827: 96; incorrect subsequent spelling of haemorrhoidalis Linnaeus, 1758.
Oestrus aemorrhoidalis Rondani, 1857: 21; incorrect subsequent spelling of haemorrhoidalis Linnaeus, 1758.

#### Selected references.

Brauer (1863: 83); Zumpt (1965: 122); Grunin (1969: 40); Pont (1973: 698); James (1974: 97); Soós and Minář (1986: 238); Cogley (1991b); Xue and Wang (1996: 2209); Otranto et al. (2005); Colwell et al. (2006: 9); Colwell et al. (2007); Zhang et al. (2016); Li et al. (2018, 2019); Yan et al. (2019).

#### Diagnosis.

Facial plate bare. Wing completely hyaline. Distance between crossveins r-m and dm-cu at least twice as long as r-m. Meron with unmodified setae. Legs yellowish brown, with femora distinctly darkened; hind tarsus with long, strong and dense setae ventrolaterally. Abdomen ground color dark brown or black. Male cercus short and broad, length/width ratio equal or less than 1.0; surstylus yellow, with an abruptly swollen lobe near base and a rounded apex; surstylar setae short, reaching at most halfway to the sagittal plane; processi longi tubercular. Female sternite 8 longitudinally ridged in the middle and with a scallop-shaped apex.

#### Material examined.

CHINA – **Inner Mongolia** • 20♂♂, 11♀♀; Chifeng, Zhaowuda League, Right Banner; 20 May–17 Sep. 1960; collector unknown; IOZ • 1♂; Ulanqab; Temurtei; 5 Jun. 1971; collector unknown; IOZ • 1♀; Xilingol League, Dongwu Banner; 24 Aug. 1971; collector unknown; IOZ. – **Heilongjiang Prov.** • 1♂; Anda; 26 Jul. 1965; collector unknown; IOZ • 1♀; Qiqihar; Fuyu County; 8 Aug. 1966; collector unknown; IOZ. – **Xinjiang Uyghur Autonomous Region** • 1♂; Wusu; 2000 m; 11 Jun. 1971; IOZ • 1♀; Kalamaili; 3 Apr. 2011; D. Zhang leg.; MBFU • 1♂; Kalamaili; 6 May 2011; D. Zhang leg.; MBFU.

#### Hosts.

Burchell’s zebra (*E. quagga burchellii*), domestic horse (*E. ferus caballus* Linnaeus), donkey (*E. africanus asinus*), Mongolian wild ass (*E. hemionus hemionus* Pallas), Mountain zebra (E. ze*bra* Linnaeus), wild horse (*E. przewalskii* Poliakov).

#### Distribution.

**Afrotropical** – Burkina Faso, Democratic Republic of the Congo, Eritrea, Ethiopia, Kenya, Namibia, Republic of the Congo, Senegal, South Africa, Sudan, Tanzania, Zambia. **Australasian** – Australia (New South Wales, Queensland, Victoria), Hawaii, New Zealand, Tasmania. **Nearctic** – Canada (Alberta, British Columbia, Manitoba, Saskatchewan), Mexico (no further data), USA (Colorado, Idaho, Illinois, Iowa, Kansas, Minnesota, Missouri, Montana, Nebraska, North Dakota, Oregon, South Dakota, Utah, Virginia, Washington, Wisconsin, Wyoming). **Neotropical** – Argentina (no further data), Venezuela. **Oriental** – India. **Palaearctic** – Austria, Azerbaijan, Belgium, Bulgaria, China (Heilongjiang, Inner Mongolia, Qinghai, Shaanxi, Tibet, Xinjiang), Czech Republic, Denmark, Finland, France (incl. Corsica), Germany, Hungary, Iran, Iraq, Italy, Kazakhstan, Kyrgyzstan, Lithuania, Malta, Mongolia, Morocco, Palestine, Poland, Romania, Russia (Tomsk, Transbaikal, Yakutsk, Yenisseisk), Slovak Republic, Sweden, Switzerland, Tajikistan, The Netherlands, Turkey, Turkmenistan, Ukraine, United Kingdom, Uzbekistan.

### Gasterophilus inermis

Taxon classificationAnimaliaDipteraOestridae

(Braurer, 1858)

9847890E-60B9-5177-A950-5ECB1622BE2D

Figs 1G–I , 4E, F , 7G–I , 10C , 11G–I , 14G–I , 17I–L ; Table 1

Gastrus inermis Brauer, 1858: 464. Type locality: Austria, Neusiedlersee, Jois (as “auf der Rossweide bei Gyois am Neusiedlersee”).

#### Selected references.

Brauer (1863: 73); Zumpt (1965: 124); Grunin (1969: 44); Soós and Minář (1986: 238); Cogley (1991b); Xue and Wang (1996: 2209); Otranto et al. (2005); Colwell et al. (2006: 36); Li et al. (2018, 2019); Yan et al. (2019).

#### Diagnosis.

Facial plate bare. Wing partly infuscate, with darkened patches with ill-defined edges. Distance between crossveins r-m and dm-cu less than length of r-m. Meron bare. Legs yellowish brown, with femora distinctly darkened. Abdomen ground color yellow. Male cercus short and broad, length/width ratio equal or less than 1.0; surstylus yellow, with a rounded apex; processi longi tubercular. Female sternite 8 longitudinally ridged in the middle and with a scallop-shaped apex.

#### Type material examined.

Syntypes of *Gastrus inermis* Brauer, 1858. AUSTRIA • 1♂, 1♀; no further data; NHMW [from photo].

#### Additional material examined.

AUSTRIA • 1♀; no locality data; 31 Jul. 1986; Waldegg leg.; NHMW [from photo] • 1♂; 1892; no further data; NHMW [from photo]. ROMANIA • 1♂, 1♀; G. Dinulescu leg.; no further data; MNHN. GERMANY • 1♂, 1♀; 1918; Wüstnei leg.; no further data; NHMD. CHINA – **Inner Mongolia** • 1♂; Chifeng, Zhaowuda League, Right Banner; 16 Aug. 1969; collector unknown; IOZ • 1♀; locality as for preceding; 22 Aug. 1969; IOZ.

#### Hosts.

Burchell’s zebra (*E. quagga burchellii*), domestic horse (*E. ferus caballus*), Mongolian wild ass (*E. hemionus hemionus*), wild horse (*E. przewalskii*).

#### Distribution.

**Afrotropical** – Senegal, South Africa. **Nearctic** – USA (Illinois). **Palaearctic** – Austria, China (Inner Mongolia, Xinjiang), Germany, Hungary, Iran, Italy, Kazakhstan, Kyrgyzstan, Romania, Mongolia, Moldova, Slovak Republic, Tajikistan, Turkmenistan, Ukraine, Uzbekistan.

#### Remarks.

Brauer (1858: 465) explicitly states that he examined “one pair” of adults that were hatched from puparia collected by the Austrian entomologist Alois Friedrich Rogenhofer in horse dung. 1♂, 1♀ in NHMW each carry two labels with the information “Oesterreich / Coll. Brauer” and “inermis / det Brauer”. A fragment of a puparium carries labels with “Gastrus / inermis / det Brauer” and “Coll. Brauer”. We consider the pair of adults to most probably represent original syntypes, but we are deliberately abstaining from designating a lectotype at this time.

### Gasterophilus intestinalis

Taxon classificationAnimaliaDipteraOestridae

(De Geer, 1776)

18F6D65A-8282-5A3B-8B4B-315E9298D246

Figs 2A–C , 5A, B , 8A–C , 10D , 12A–C , 15A–C , 17M–P ; Table 1

Oestrus intestinalis De Geer, 1776: 292. Type locality: Sweden.
Oestrus equi Clark, 1797: 298. Junior primary homonym of Oestrus equi Fabricius, 1787. Type locality: England.
Oestrus gastricus major Schwab, 1840: 31. Unavailable name; proposed in synonymy with Oestrus intestinalis De Geer, 1776 and Oestrus equi Clark, 1797 and not made available from subsequent use as a valid name for a taxon before 1961.
Oestrus bengalensis Macquart, 1843: 182. Type locality: Bangladesh (as “Du Bengal”) and India.
Oestrus gastrophilus Gistel, 1848: 153 (as “O. gastrophilus, mihi. O. Equi. Linné.”). Type locality: not given, probably Germany.
Oestrus schwabianus Gistel, 1848: 153 (as “Oestrus Schwabianus, mihi. O. gastric. major Schwab”). Type locality: not given, probably Germany, Bavaria.
Gastrophilus equi var. asininus Brauer, 1863: 71. Type locality: Egypt and Sudan (“Egypten” & “Nubien”).
Gastrophilus aequi : Brauer 1863: 28; incorrect subsequent spelling of equi Clark, 1797.
Gasterophilus magnicornis Bezzi, 1916: 29. Type locality: Eritrea.

#### Selected references.

Zumpt (1965: 125); Grunin (1969: 48); Pont (1973: 698); James (1974: 96); Kettle (1974); Soós and Minář (1986: 238); Cogley (1991b); Escartin and Bautista (1993); Xue and Wang (1996: 2210); Otranto et al. (2005); Colwell et al. (2006: 4); Colwell et al. (2007); Felix et al. (2007); Güiris et al. (2010); Ganjali and Keighobadi (2016); Zhang et al. (2016); Li et al. (2018); Yan et al. (2019).

#### Diagnosis.

Facial plate bare. Wing partly infuscate, with darkened patches with ill-defined edges; crossvein dm-cu situated almost opposite of crossvein r-m. Meron with unmodified setae. Legs yellow, with more or less dark coloration on tarsus; hind trochanter with a spatulate process in male and a tubercle in female. Abdomen ground color yellow in both male and female. Male cercus elongated and broad, length-width ratio around 1.5; surstylus mainly yellow with black coloration apically, and a rounded apex; processi longi elongated. Female abdominal segment 7 distinctly longer than broad, sternite 8 longitudinally ridged in the middle and with a scallop-shaped apex.

#### Material examined.

CHINA • – **Inner Mongolia** • 13♂♂, 26♀♀; Chifeng; Zhaowuda League, Right Banner; 13 Jun.–17 Sep. 1960; collector unknown; IOZ • 1♀; Hulunbeir; Genhe; 13 Aug. 1971; collector unknown; IOZ • 1♀; Hulunbeir; Yakeshi; 19 Aug. 1971; collector unknown; IOZ • 1♀; Hailaer; 23 Aug. 1971; collector unknown; IOZ • 1♀; Hulunbeir; Yakeshi; Boketu; 28 Aug 1971; collector unknown; IOZ • 1♀; Ulanqab; Temurtei; 29 Aug. 1971; collector unknown; IOZ • 7♂♂; Ulanqab; Temurtei; 29 Aug. 1971; collector unknown; IOZ • 2♀♀; Ulanqab, Temurtei; 30 Aug. 1971; collector unknown; IOZ. – **Heilongjiang Prov.** • 2♂♂, 2♀♀; Anda; 26–27 Aug. 1965; collector unknown; IOZ • 3♀♀; Qiqihar, Fuyu County; 15 Jun.–26 Aug. 1966; collector unknown; IOZ • 1♀; Daqing, Lamadian County; 15 Aug. 1969; collector unknown; IOZ • 1♀; locality as for preceding; 17 Sep. 1969; collector unknown; IOZ • 1♀; Qiqihar, Tailai County, Jiangning; 20 Jun. 1970; collector unknown; IOZ • 5♂♂, 1♀; Mudanjiang, Ning’an; 2 Sep. 1970; collector unknown; IOZ. – **Beijing** • 1♀; Yanqing County; 4 Aug. 1970; collector unknown; IOZ. – **Tibet Autonomous Region** • 1♂; Xinglin; 2550 m; 18 Aug. 1974; collector unknown; IOZ. – **Sichuan Prov.** • 1♀; Aba Autonomous Prefecture, Hongyuan County; 3700 m; 27 Aug. 1983; collector unknown; IOZ • 1♀; locality as for preceding; 3500 m; 28 Aug. 1983; collector unknown; IOZ • 1♂, 4♀♀; Ruoergai County; 30 Aug.–1 Sep. 1983; collector unknown; IOZ. • 1♀; no further data; MNHN.

#### Hosts.

Domestic horse (*E. ferus caballus*), donkey (*E. africanus asinus*), Mongolian wild ass (*E. hemionus hemionus*), wild horse (*E. przewalskii*).

#### Distribution.

**Afrotropical** – Burkina Faso, Chad, Eritrea, Ethiopia, Ghana, Kenya, Morocco, Nigeria, Republic of the Congo, Senegal, South Africa, Sudan, Tanzania. **Australasian** – Australia (New South Wales, Norfolk I, Tasmania), Hawaii, New Zealand. **Nearctic** – Canada (Alberta, British Columbia, Manitoba, New Brunswick, Ontario, Quebec, Saskatchewan), Mexico (Aguascalientes, Chiapas), USA (Arizona, California, Colorado, Connecticut, Idaho, Illinois, Iowa, Kansas, Maine, Maryland, Massachusetts, Michigan, Minnesota, Mississippi, Missouri, Montana, Nebraska, New Hampshire, New Jersey, New Mexico, New York, North Carolina, North Dakota, Ohio, Oklahoma, Oregon, South Dakota, Texas, Utah, Vermont, Virginia, Washington, Wisconsin, Wyoming). **Neotropical** – Argentina (no further data), Brazil (Rio Grande do Sul), Chile (Bío Bío Region), Jamaica, Venezuela. **Oriental** – India. **Palaearctic** – Bangladesh, Belgium, China (Beijing, Gansu, Heilongjiang, Inner Mongolia, Qinghai, Shanxi, Shaanxi, Sichuan, Tibet, Xinjiang, Yunnan), Czech Republic, Denmark, Egypt, Finland, France (incl. Corsica), Germany, Hungary, Ireland, Iran, Italy (incl. Sicily), Jordan, Lithuania, Mongolia, Norway, Pakistan, Poland, Romania, Slovak Republic, Sweden, Switzerland, The Netherlands, Turkey, Ukraine, United Kingdom.

### Gasterophilus meridionalis

Taxon classificationAnimaliaDipteraOestridae

(Pillers & Evans, 1926)

37D79E21-9A86-5CF7-818C-C0A5EBA509A5

Figs 2D–F , 5C, D , 11E , 15D–F ; Table 1

Oestrus meridionalis Pillers & Evans, 1926: 264. Type locality: Zimbabwe (as “Rhodesia”).

#### Selected references.

Zumpt (1965: 121); Cogley (1991b); Colwell et al. (2006: 36); Colwell et al. (2007: 256).

#### Diagnosis.

Male unknown. Antennal postpedicel long-oval. Facial plate setose. Wing completely hyaline. Crossvein dm-cu extremely weak, with only a faint trace; distance between crossveins r-m and dm-cu equal or less than length of r-m. Meron with unmodified setae. Legs black or black-brown. Abdomen ground color dark brown. Female sternite 8 longitudinally ridged in the middle and with a scallop-shaped apex.

#### Material examined.

SOUTH AFRICA • 2♀♀; Transvaal; Newington; 15 Aug. 1957; reared from third instar larvae by F. Zumpt; KZNM.

#### Hosts.

Burchell’s zebra (*E. quagga burchellii*).

#### Distribution.

**Afrotropical** – Botswana, Democratic Republic of the Congo, Mozambique, Namibia, Republic of the Congo, South Africa, Tanzania, Zambia, Zimbabwe.

### Gasterophilus nasalis

Taxon classificationAnimaliaDipteraOestridae

(Linnaeus, 1758)

87B35415-7F0F-543F-B371-B9B906EFB8E7

Figs 2G–I , 5E, F , 8D–F , 10F , 11F , 12D–F , 15G–I ; Table 1

Oestrus nasalis Linnaeus, 1758: 584. Type locality: Sweden (through reference to *Fauna Svecica*).
Oestrus equi Fabricius, 1787: 321. Type locality: not given, probably Europe.
Oestrus veterinus Clark, 1797: 312. New replacement name for Oestrus nasalis Linnaeus, 1758 [“I have given it the name of veterinus .... in preference to the erroneous one of nasalis” (p. 313)].
Oestrus salutaris Clark, 1815: pl. 1. Nomen nudum.
Gasterophilus clarkii Leach, 1817: 2. Type locality: England, Bantham close to Kingsbridge (as “Habitat in Anglia Occidentali. Apud Bantham prope Kingsbridge a meipso captus”).
Gastrus jumentarum Meigen, 1824: 179. Type locality: not given, probably Denmark (as “Ein Weibchen in dem Koppenhagener Königl. Museum”).
Oestrus gastricus minor Schwab, 1840: 40. Unavailable name proposed in synonymy with Oestrus nasalis Linnaeus, 1758 and Oestrus veterinus Clark, 1797 and not made available from subsequent use as a valid name for a taxon before 1961.
Gastrus subjacens Walker, 1849: 687. Type locality: Canada, Nova Scotia.
Oestrus stomachinus Gistel, 1848: 153. Type locality: not given, probably Germany, Bavaria.
Gasterophilus crossi Patton, 1924: 963. Type locality: India, Punjab.
Gastrophilus albescens Pleske, 1926: 228. Type locality: Egypt, Cairo (as “Il provient de l’Egypte des environs du Caire”).
Gastrophilus nasalis var. nudicollis Dinulescu, 1932: 28, 32. Type locality: not given.
Gastrophilus veterinus var. aureus Dinulescu, 1938: 315. Type locality: not given.
Gastrus jumentorum : Brauer 1863: 87, 280; incorrect subsequent spelling of jumentarum Meigen, 1824.
Oestrus nasulis : Fabricius 1787: 321; incorrect subsequent spelling of nasalis Linnaeus, 1758.

#### Selected references.

Zumpt (1965: 117); Grunin (1969: 32); Pont (1973: 698); Kettle (1974); Soós and Minář (1986: 238); Cogley (1991b); Escartin and Bautista (1993); Xue and Wang (1996: 2210); Sequeira et al. (2001); Otranto et al. (2005); Colwell et al. (2006: 6); Colwell et al. (2007); Felix et al. (2007); Zhang et al. (2016); Li et al. (2018); Yan et al. (2019).

#### Diagnosis.

Facial plate setose. Wing entirely hyaline; distance between crossveins r-m and dm-cu less than length of r-m. Meron with unmodified setae. Legs mainly black-brown. Abdomen ground color dark brown or black, with reddish-yellow hair-like setae on tergites 5–7 in male, pale yellow in female. Male cercus long and narrow, length/width ratio more than 3.0; surstylus yellow, with gradually a tapered apex; processi longi elongated and distinctly bent inwards. Female sternite 8 longitudinally ridged in the middle and with flattened and a scallop-shaped apex.

#### Type material examined.

Holotype of *Gastrophilus albescens* Pleske, 1926. EGYPT • ♂; Cairo; no further information; ZIN.

#### Additional material examined.

CHINA – **Inner Mongolia** • 2♂♂, 5♀♀; Chifeng; Zhaowuda League, Right Banner; 24 May–10 Aug. 1960; collector unknown; IOZ • 5♂, 1♀; Ulanqab, Temurtei County; 12–30 Aug. 1971; Y.R. Zhang leg.; IOZ. – **Xinjiang Uyghur Autonomous Region** • 1♂; Altay, Qinghe County; 6 Jul. 1960; S.Y. Wang leg.; IOZ • 1♀; Altyn-Tagh; 3850 m; 7 Aug. 1988; X.Z. Zhang leg.; IOZ • 1♂; locality as for preceding; 11 Aug. 1988; X.Z. Zhang leg.; IOZ • 3♂♂; Fuyun County; Qiakuertu; 25 May–3 Jun. 2010; F. Mo leg.; MBFU • 8♂♂, 1♀; Kalamaili; 18 Apr.–25 Jun. 2010; D. Zhang leg.; MBFU • 1♂, 5♀♀; Kalamaili; 16 Apr.–8 May 2011; D. Zhang leg.; MBFU.

#### Hosts.

Burchell’s zebra (*E. quagga burchellii*), domestic horse (*E. ferus caballus*), donkey (*E. africanus asinus*), Mongolian wild ass (*E. hemionus hemionus*), wild horse (*E. przewalskii*).

#### Distribution.

**Afrotropical** – Burkina Faso, Ethiopia, Kenya, Lesotho, Morocco, Namibia, Senegal, South Africa, Zambia, Zimbabwe. **Australasian** – Australia (Queensland, Tasmania), Fiji, Hawaii, New Zealand. **Nearctic** – Canada (Alberta, British Columbia, Manitoba, Northwestern, Nova Scotia, Quebec, Saskatchewan), Mexico (Aguascalientes, San Vicente Chicoloapan), USA (Arizona, California, Colorado, Illinois, Iowa, Kansas, Kentucky, Maryland, Michigan, Minnesota, Missouri, Montana, Nebraska, New Jersey, New Mexico, New York, North Dakota, Ohio, Oklahoma, Oregon, South Dakota, Texas, Washington, Wyoming). **Neotropical** – Antigua and Barbuda, Argentina, Brazil (Rio Grande do Sul, São Paulo), Chile (Bío Bío Region), Jamaica, Panama, Puerto Rico, Uruguay, Venezuela. **Oriental** – India, Malaysia, Myanmar, Thailand. **Palaearctic** – Afghanistan, Austria, Bulgaria, China (Heilongjiang, Inner Mongolia, Shaanxi, Tibet, Xinjiang), Cyprus, Denmark, Egypt, France, Germany, Hungary, Iraq, Italy (incl. Corsica and Sicily), Jordan, Kazakhstan, Kyrgyzstan, Lithuania, Mongolia, Morocco, Pakistan, Poland, Romania, Russia (Tomsk), Sweden, Switzerland, The Netherlands, Tajikistan, Turkey, Turkmenistan, Ukraine, United Kingdom, Uzbekistan.

### Gasterophilus nigricornis

Taxon classificationAnimaliaDipteraOestridae

(Loew, 1863)

CF008C0A-4380-5D3C-B0DF-FC41FD3D026C

Figs 3A–C , 6A, B , 9A–C , 10G , 13A–C , 16A–C , 18E–H ; Table 1

Gastrus nigricornis Loew, 1863: 38. Type locality: Moldova, Bessarabia (as “Bessarabien”).
Gastrophilus viridis Sultanov, 1951: 41. Type locality: Kazakhstan, Kzyl-Ordinskaja, around Teren-Uzyakaskiy.
Gasterophilus migricornis : Colwell 2006: 291; incorrect subsequent spelling of nigricornis Loew, 1863.

#### Selected references.

Zumpt (1965: 119); Grunin (1969: 36); Soós and Minář (1986: 239); Xue and Wang (1996: 2214); Colwell et al. (2006: 36); Zhang et al. (2012, 2016); Li et al. (2018); Yan et al. (2019).

#### Diagnosis.

Antennal postpedicel red-brown to blackish. Facial plate setose. Meral setae with swollen tip. Wing completely hyaline. Crossvein dm-cu absent. Legs yellowish brown with femora distinctly darkened. Male cercus long and narrow, length/width ratio more than 3.0; surstylus yellow, with a rounded apex; processi longi elongated. Female sternite 8 longitudinally ridged in the middle and with a scallop-shaped apex.

#### Material examined.

CHINA – **Xinjiang Uyghur Autonomous Region** • 1♂; Barköl Kazak Autonomous County, Saerqiaoke; 14 Aug. 1968; collector unknown; IOZ • 9♂♂, 1♀; Kalamaili; 3 Apr.–18 May 2009; D. Zhang leg.; MBFU • 27♂♂, 1♀; Fuyun County, Qiakuertu; 25 Apr.–13 May 2009; F. Mo leg.; MBFU.

#### Hosts.

Domestic horse (*E. ferus caballus*), donkey (*E. africanus asinus*), Mongolian wild ass (*E. hemionus hemionus*), wild horse (*E. przewalskii*).

#### Distribution.

**Palaearctic** – China (Inner Mongolia, Qinghai, Xinjiang), Kazakhstan, Kyrgyzstan, Moldova, Mongolia, Tajikistan, Turkmenistan, Ukraine, Uzbekistan.

#### Remarks.

The distribution of *G. nigricornis* appears to be limited to far eastern Europe and Central Asia. Thus, reports of *G. nigricornis* from western part of Europe [Spain: Lucientes (2002); Italy: Pape (2013)] are suspected to be misidentifications and the records are not included.

### Gasterophilus pecorum

Taxon classificationAnimaliaDipteraOestridae

(Fabricius, 1794)

42BE358C-0E7B-5E2D-AAAE-B5014FD931C6

Figs 3D–F , 6C, D , 9D–F , 10H , 13D–F , 16D–F , 18I–L ; Table 1

Oestrus pecorum Fabricius, 1794: 230. Type locality: not given, probably Europe.
Oestrus vituli Fabricius, 1794: 231. Type locality: not given, but at least Sweden and France by reference to works of Linnaeus and Geoffroy.
Gastrus jubarum Meigen, 1824: 179, 180. Type locality: Austria.
Gastrus lativentris Brauer, 1858: 465. Type locality: Latvia, Curland (as “in Kurland gefangen”).
Gastrus ferruginatus Zetterstedt, 1844: 978. Type locality: Sweden, Skåne, Tranås socken, Esperöd. (as “ad Esperöd in parœcia Tranås Scaniæ”).
Gasterophilus pecorum var. zebrae Rodhain & Bequaert, 1920: 181. Type locality: Kenya and Tanzania.
Gastrophilus vulpecula Pleske, 1926: 227. Type locality: China, Inner Mongolia, Alxa League.
Gastrophilus gammeli Szilády, 1935: 140. Type locality: Hungary.
Gastrophilus hammeli : Paramonov 1940: 34, 46; incorrect subsequent spelling of gammeli Szilády, 1935.
Gastrophilus hummeli : Paramonov 1940: 32; incorrect subsequent spelling of gammeli Szilády, 1935.
Gastrus selysi Walker, 1849: 687. Nomen nudum.

#### Selected references.

Zumpt (1965: 114); Grunin (1969: 25); Pont (1973: 698); Soós and Minář (1986: 239); Cogley (1991b); Xue and Wang (1996: 2210); Otranto et al. (2005); Colwell et al. (2006: 5); Colwell et al. (2007); Zhang et al. (2016); Hoseini et al. (2017); Li et al. (2018); Yan et al. (2019).

#### Diagnosis.

Antennal pedicel elongated, with length/width ratio more than 0.8. Facial plate setose. Wing dark, with broad darkened patches with ill-defined edges; crossvein dm-cu absent. Meron with unmodified setae. Legs yellowish brown with femora distinctly darkened. Abdomen ground color yellow in male, mainly dark brown to black in female. Male cercus long and narrow, length/width ratio more than 3.0; surstylus yellow, with a rounded apex; processi longi elongated. Female sternite 8 with a longitudinal concavity in the middle and with a keel-shaped apex.

#### Material examined.

CHINA – **Xinjiang Uyghur Autonomous Region** • 1♂; Akesu; 25 Sep. 1958; collector unknown; IOZ • 1♀; Bayingolin Mongol Autonomous Prefecture, Qiemo County; Aqiang; 3000 m; 20 Jul. 1988; X.Z. Zhang leg.; IOZ • 2♀♀; Fuyun County, Qiakuertu; 8–10 Jun. 2009, F. Mo leg.; MBFU • 9♂♂, 4♀♀; Kalamaili; 6 May–1 Jun. 2009; D. Zhang leg.; MBFU. – **Inner Mongolia** • 1♀; Chifeng, Zhaowuda League, Right Banner; 22 Aug.–28 Sep. 1959; collector unknown; IOZ • 2♂♂; Ulanqab, Temurtei, 4–27 Aug. 1971; collector unknown; IOZ • 1♂; Xisuqi; 1 Sep. 1971; collector unknown; IOZ.

#### Hosts.

Burchell’s zebra (*E. quagga burchellii*), domestic horse (*E. ferus caballus*), donkey (*E. africanus asinus*), Mongolian wild ass (*E. hemionus hemionus*), Persian onager (*E. hemionus onager* Boddaert), wild horse (*E. przewalskii*).

#### Distribution.

**Afrotropical** – Burkina Faso, Kenya, Namibia, Senegal, South Africa, Tanzania, Uganda, Zambia. **Oriental** – India. **Palaearctic** – Austria, Belgium, China (Heilongjiang, Inner Mongolia, Xinjiang), Czech Republic, Denmark, France, Germany, Hungary, Iran, Italy (incl. Corsica and Sicily), Latvia, Lithuania, Mongolia, Poland, Romania, Sweden, Switzerland, The Netherlands, Turkey, Ukraine, United Kingdom.

### Gasterophilus ternicinctus

Taxon classificationAnimaliaDipteraOestridae

(Gedoelst, 1912)

41CF737E-A4E3-5C2F-B057-94CBF6408298

Figs 3G–I , 6E, F , 9G–I , 10I , 13G–I ; Table 1

Gasterophilus ternicinctus Gedoelst, 1912: 426. Type locality: Democratic Republic of the Congo (as “Zaire”), 11.5 km W of Luapula river (as “6 milles W. du Luapula”).
Gasterophilus gedoelsti Rodhain & Bequaert, 1920: 188. Type locality: Kenya.

#### Selected references.

Zumpt (1965: 128); Cogley (1991b); Colwell et al. (2006: 36).

#### Diagnosis.

Facial plate bare. Wing with darkened patches with demarcated edges. Distance between crossveins r-m and dm-cu less than length of r-m. Meron with unmodified setae. Legs yellowish brown, with tibiae and tarsi more or less darkened. Hind trochanter of male with a long, spatulate process, of female with a tubercle; hind tibia and tarsus flattened distinctly in male, slightly in female; tarsomeres 2–4 shortened in both sexes, broader than long. Abdomen ground color yellow in both male and female. Male cercus elongated and broad, length/width ratio around 1.5; surstylus mainly black with yellow coloration basally, and a rounded apex. Female abdominal segment 7 distinctly longer than broad, sternite 8 longitudinally ridged in the middle and with a scallop-shaped apex.

#### Material examined.

SOUTH AFRICA • 1♂; KwaZulu; Hluhluwe-Imfolozi Park; 8 Mar. 1963; collector unknown; MBFU. KENYA • 1♂, 1♀; Kenplains, Athi river; 13 Mar. 1991; C.F. Dewhurst leg.; NHMUK.

#### Hosts.

Burchell’s zebra (*E. quagga burchellii*).

#### Distribution.

**Afrotropical** – Burkina Faso, Democratic Republic of the Congo, Kenya, Republic of the Congo, Senegal, South Africa, Zambia.

## Supplementary Material

XML Treatment for Gasterophilus

XML Treatment for Gasterophilus flavipes

XML Treatment for Gasterophilus haemorrhoidalis

XML Treatment for Gasterophilus inermis

XML Treatment for Gasterophilus intestinalis

XML Treatment for Gasterophilus meridionalis

XML Treatment for Gasterophilus nasalis

XML Treatment for Gasterophilus nigricornis

XML Treatment for Gasterophilus pecorum

XML Treatment for Gasterophilus ternicinctus
